# Reproductive age mortality survey (RAMOS) in Accra, Ghana

**DOI:** 10.1186/1742-4755-6-7

**Published:** 2009-06-04

**Authors:** Afisah Yakubu Zakariah, Sophie Alexander, Jos van Roosmalen, Pierre Buekens, Enyonam Yao Kwawukume, Patrick Frimpong

**Affiliations:** 1 Disease Control and Prevention Department, Public Health Division, GHS/MOH, Accra, Ghana; 2 Unité Santé Reproductive et Epidémiologie Périnatale, Ecole de Santé Publique, Université Libre de Bruxelles, Brussels, Belgium; 3 Leiden University Medical Centre, Leiden, the Netherlands; 4 VU University Medical Centre, Amsterdam, the Netherlands; 5 School of Public Health and Tropical Medicine, Tulane University, New Orleans, Lousiana, USA; 6 Korle-Bu Teaching Hospital, Accra, Ghana; 7 La General Hospital, Accra, Ghana

## Abstract

**Background:**

Maternal mortality remains a severe problem in many parts of the world, despite efforts to reach MDG 5. In addition, underreporting is an issue especially in low income countries. Our objective has been to identify the magnitude of maternal deaths and the degree of underreporting of these deaths in Accra Metropolis in Ghana during a one year period.

**Methods:**

A Reproductive Age Mortality survey (RAMOS) was carried out in the Accra Metropolis for the period 1st January 2002-31st December 2002. We reviewed records of female deaths aged 10–50 years in the Metropolis for the whole year 2002 using multiple sources. Maternal deaths identified through the review were compared with the officially reported maternal deaths for the same period.

**Results:**

At the end of the study, a total of 179 maternal deaths out of 9,248 female deaths between the ages of 10–50 years were identified. One hundred and one (N = 101) of these were reported, giving an underreporting rate of 44%. The 179 cases consisted of 146 (81.6%) direct maternal deaths and 32 (17.9%) indirect maternal deaths and 1 (0.6%) non maternal death. The most frequent causes of direct maternal deaths were obstetric haemorrhage (57; 32%), pregnancies with abortive outcome (37; 20.8%), (pre) eclampsia (26; 14.6%) and puerperal sepsis (13; 7.3%). The most frequent indirect cause was sickle cell crisis in pregnancy (13; 7.3%).

**Conclusion:**

A Reproductive Age Mortality Survey is an effective method that could be used to update data on maternal mortality in Ghana while efforts are made to improve on maternal death audits in the health facilities. Strengthening the existing community based volunteers to report deaths that take place at home and the civil registration systems of births and deaths is also highly recommended.

## Background

The complexity of ascertaining maternal deaths makes it difficult for many low income countries to measure the levels of maternal mortality, hence the lack of valid data on such avoidable tragedies [1]. Even high income countries with complete registration systems of births and deaths find it difficult to report all maternal deaths [2-4]. However, this should not deter resource poor countries from improving on the quality of ascertainment of maternal deaths. Different approaches such as community-based maternal deaths reviews (verbal autopsies), facility based maternal deaths reviews, reproductive age mortality surveys (RAMOS), and confidential enquiries into maternal deaths are used to ascertain maternal deaths. These approaches are very effective and have been developed mainly for countries where levels of maternal mortality are high. Maternal death audit for resource-poor countries could be more feasible at the facility level than the community level [5]. These methods could be used to provide data on maternal mortality and the cause of death.

In an earlier study, a retrospective review of available sources of information on maternal deaths in Greater Accra Region, Ghana for the year 2000 was carried out. Analysis of the civil registers of causes of death for the region identified 127 maternal deaths, which was double the officially reported figure (n = 63) for the same year. Further review of the records of four major hospitals offering complete obstetric care services in the same region for the same time period identified 148 maternal deaths. This figure is almost three times higher than the officially reported one for the same year [6]. These findings indicated under-reporting of maternal deaths in the official statistics by using only institutional data and hence prompted the present study.

In this study, we conducted a reproductive age mortality survey (RAMOS) for the year 2002 using multiple sources approach in identifying maternal deaths. Multiple sources techniques are now being increasingly employed to correct for under reporting of cases in epidemiological studies [5-7]. In this study, we aimed at identifying the magnitude of maternal deaths in the city and to identify the degree of underreporting using reported institutional data.

## Methods

### Identification of Maternal deaths

This study is part of a confidential enquiry into maternal deaths undertaken in Accra city for the period, 1st January 2002-31st December 2002 and pertains specifically to completeness of reporting.

A reproductive age mortality survey was carried out in the Accra Metropolis of Ghana. We identified all possible deaths among women of reproductive age between 10 to 50 years of age using multiple sources to identify the cause of death and ways to prevent such deaths. We reviewed medical records (admission and discharge books, death certificate books, death registers, mortuary logbooks and individual case notes when necessary) of females aged 10–50 years who died in these health facilities as well as women who died outside the hospitals, but were brought to the mortuary, being a legal requirement for all deaths in Accra.

In Korle-Bu and 37 Military Hospitals in the Accra Metropolis, Pathologists carry out post mortem examinations. In deaths that occur outside any health facility, or deaths that occur less than 24 hours after reaching a health care facility, post mortem examination is carried out in Accra to establish the cause of death before permission for burial is given. The Pathologist enters the findings into the mortuary log books including information about deaths taking place within and outside hospital environment in Accra and diagnosis and post mortem report.

Maternal deaths were identified according to ICD-10 definition of a maternal death [8]. All pregnancy related deaths were included. The definitions used in identifying and classifying the cases are as follows:

• "A maternal death is defined as the death of a woman while pregnant or within 42 days of termination of pregnancy, irrespective of the duration and site of the pregnancy, from any cause related to or aggravated by the pregnancy or its management but not from accidental or incidental causes". ICD 10 definition of a maternal death includes pregnancy related deaths* and late maternal deaths**

• *Pregnancy-related Death: The death of a woman while pregnant or within 42 days of termination of pregnancy, irrespective of the cause of death (ICD-10)

• **Late maternal death: The death of a woman from direct or indirect obstetric cause more than 42 days but less than one year after termination of pregnancy

• **Direct maternal death**: Is the death of a woman as result of a complication of the pregnancy, delivery, or their management

• **Indirect maternal death: **Those resulting from previous existing disease or disease that developed during pregnancy and that was not due to direct obstetric cause but was aggravated by the physiological effects of pregnancy

• **Incidental deaths: **Other fatalities during but unrelated to a pregnancy. They are also termed non obstetrical maternal deaths

Variables that were recorded included: name, age, place of birth, date of death, place of birth, diagnosis, pregnancy and childbirth status (Last menstrual period, delivery), cause of death (by Clinician and Pathologist.

Data on maternal deaths that were prospectively reported and documented in all twelve government and quasi government health facilities (one Teaching hospital, four hospitals and 7 Polyclinics) as well as the five public mortuaries in the metropolis during the study period were collected.

The maternal deaths identified through RAMOS were compared with those actively reported and documented prospectively for the same period. They were matched by name, age, cause of death, date and place of death for underreporting.

An Internal Expert Committee was set up. This committee comprised of two obstetricians from Accra who classified the deaths into direct, indirect and pregnancy related deaths. The deaths that took place outside health facilities were classified using the cause of death stated in the mortuary logbooks assigned by the Pathologists.

## Results

A total number of 9,248 health care records of women in their reproductive age (10–50 years) in a population of approximately 2,905,726 inhabitants were reviewed during the study period. Among those, a total of 179 women died while pregnant or within 42 days of termination of a pregnancy. One hundred and one (N = 101; 56%) of the identified maternal deaths were also found in the prospective reporting system, which had been established for the study, giving an underreporting of 44% (table 1). We could not identify cases of late maternal deaths.

**Table 1:** Identified maternal deaths in Accra City, Ghana 2002

Method of identification of Maternal deaths	Number of Maternal deaths	Percentage
Identified maternal deaths through RAMOS	179	100
Officially reported Deaths	101	56
**Underreported**	**78**	**44**

### Underlying causes of maternal deaths

The 179 cases consisted of 146 (81.6%) direct maternal deaths, 32 (17.9%) indirect maternal death and 1 (0.6%) non maternal death. The most frequent causes of direct maternal deaths were obstetric haemorrhage (57; 32%), pregnancies with abortive outcome (37; 20.8%), (pre) eclampsia (26; 14.6%) and puerperal sepsis (13; 7.3%). The most frequent indirect cause was sickle cell crisis in pregnancy (13; 7.3%) (table 2).

**Table 2:** Causes of maternal deaths

ICD 10 Classification	Number	Percentage
***Direct maternal deaths***		
Obstetric haemorrhage	57	32
(Pre) eclampsia	26	14.6
Genital tract sepsis	13	7.3
Pregnancies with abortive outcome	37	20.8
Other direct causes	13	7.3
**Total**	**146**	**82**
		
***Indirect causes***		
Sickle Cell with complications	13	7.3
Pneumonia	4	2.2
Anaemia	2	1.1
HIV/AIDS	2	1.1
Hepatitis	2	1.1
Cerebrovascular disease	2	1.1
Others*	7	3.9
**Total**	**32**	**18**
		
**Total direct and indirect maternal deaths**	**178**	**100**

*Other indirect causes include: cervical cancer (n = 2), jaundice (n = 1), cerebral oedema (n = 1), congestive cardiac failure (n = 1), bacterial endocarditis (n = 1) and pericarditis (n = 1)

### Demographic characteristics

The mean age of the women who died was 29 years ± 6.4; minimum age was 15 years and maximum 49 years. There were no maternal deaths identified among the age group 10–14 years. One hundred and forty one of the identified maternal deaths were between the ages of 15–34 years, and 38 cases were 35 years and above. The mean parity was 1.9 ± 1.8 and the range 0–8.

Out of the 179 identified deaths, 79 of them spent more than 24 hours on admission into a hospital before death, 28 spent less than 24 hours on admission and 72 of them either died at home or in an unspecified health facility. All 79 women that spent more than 24 hours on admission into hospital before death were reported by the health facilities, and 6/28 of those who died less than 24 hours after admission to a health facility were reported. None of the 72 cases that either died at home or in an unspecified health facility were captured prospectively and reported. The cases that spent less than 24 hours after admission and those who died outside health facilities are referred to as "coroners' cases". They require additional police investigation into the cause of death (table 3).

**Table 3:** Method of identification of maternal deaths and Length of stay on admission to a health facility before death

Method of identification of Maternal deaths	*More than 24 hours of admission into a hospital	**Less than 24 hours of admission into a hospital	**Either died at home or in unspecified health facility	Total
RAMOS	79	28	72	179
Officially reported Deaths	79	22	0	101
**Underreported**	**0**	**6**	**72**	**78**

*Cases that spent more than 24 hours on admission are referred to as non-coroners' cases

**Those that spent less than 24 hours on admission or either died at home are referred to as coroners' cases. They require a police investigation into the cause of death.

A post mortem examination was performed on 125 cases (69.8%).

## Discussion

Maternal mortality is grossly underestimated in the country given that almost fifty percent of the deaths related to pregnancy were underreported.

The figures found in our Reproductive Age Mortality Survey almost doubled when compared to those of the officially reported figures. Our results highlight the importance of ascertainment of maternal deaths by using multiple sources [5-7,9-12]. Based on the fact that 44% of unreported cases were identified in the capital city, which presumably has the best registration system of births and deaths in the country, underreporting is a true cause for concern, and may be markedly worse in other parts of the country that do not have any system of reporting and registering of deaths [6]. Our results underline the persistence of underreporting of maternal deaths, as mentioned by many authors, both in industrialized [2-4] and in low income countries [6,10-12].

The present data can be compared with other available data. The 2000 Annual Report on Reproductive and Child Health by the Ministry of Health of Ghana indicated 110 maternal deaths/100.000 live births for the Greater Accra Region in the year 2000 [13]. However, despite the higher estimate of mortality ratio for Accra city during the study period, this figure is much lower than the published maternal mortality ratio for the Greater Accra Region for the year 2000 [6] and estimates from other parts of the country, as well as ratios obtained from the sub-region [14-20]. The observed differences in ratios could be explained, at least in part by the study design. The other studies present mostly hospital-based derived maternal mortality ratios. Our study could be considered almost population based because it included all government health facilities as well as the five public mortuaries in the metropolis with the exception of private health facilities which form a very small fraction of health care facilities in the metropolis. However, deaths taking place in any of these private health facilities pass through one of these 5 mortuaries in the city before the body is buried.

Though our technique nearly doubled the reported number of deaths, it remains to be discussed how many might have escaped the mortuary logbooks and other available sources of information used for data collection. We would contend that it is unlikely to be a frequent event. Though it is generally accepted that in rural areas women may die and be buried on their own land without any form of notification, this is unlikely to happen on a large scale in the Accra Metropolis area. It is possible that on some occasions, after a maternal death during an unattended home delivery, the corpse may have been transported out of town to a country residence and buried there. However, we believe that such events, even if they occurred, were exceptional. With regard to the exhaustive nature of the data collection, most deaths that occurred in the health institutions and at home were identified. Our study included all the main public reference maternity units, primary and secondary maternity units, as well as all mortuaries in the city. We therefore assume that a large majority of the maternal deaths have been identified. However, we cannot tell, among the remaining deaths of women in their reproductive age, the number of cases that might have been missed due to misclassification [3].

In our study, we identified a significant relationship between the prospective reporting system and the length of stay in hospital before death, as well as the performance of a post mortem examination. The cases that were admitted into health facilities more than 24 hours before dying were more likely to be identified and reported since the underlying cause of death would be known. Hence there would neither be the need for a post mortem examination nor police investigation to identify the cause of death. The reported cases died in health facilities with an assigned cause of death being maternal. Such cases are more easily reported than those dying outside a hospital environment with an unknown cause of death. In these, a post mortem examination needs to be performed to identify the cause of death. Such cases do not appear in the hospital admission and discharge books but appear only in the mortuary log-books and this could lead to underreporting of cases. Furthermore, in areas where there are no Pathologists to carryout post mortem examination on deaths taking place outside the hospital environment, such deaths would not be able to be classified and would lead to gross underreporting of cases.

The limitations of our approach are that there still is a possibility that ascertainment is incomplete because of possible omission of pregnancy or childbirth status in mortuary logbooks which qualifies it to be classified as maternal death. The other limitation is that our approach relied on the fact that (virtually all) women in Accra are buried, and need a death certificate. A similar study in rural areas would be very difficult to set up.

Computing a maternal mortality ratio using our approach (RAMOS) captured more deaths than the routine reporting. However, this did not provide adequate information on live births.

## Conclusion

Measuring maternal mortality accurately is complex and expensive; hence the reasons that resource poor countries have difficulty in providing accurate figures.

A Reproductive Age Mortality Survey is, in our experience, an effective method that is able to identify deaths that otherwise would have been missed relying solely on officially reported institutional maternal deaths. However, notification of maternal deaths in the various institutions still needs improvement. This could be used to update data on maternal mortality in Ghana while efforts are made to improve on the civil registration system of births and deaths as well as certification of deaths in Ghana.

Maternal death audits in the health facilities should be encouraged to function properly as well as strengthening the existing community based volunteers to report deaths that take place at home.

In hospitals where there are pathologists, they need to get involved actively in the reporting of maternal death since they capture cases outside the health facility environment as well.

## Competing interests

The authors declare that they have no competing interests.

## Authors' contributions

AYZ was involved in the design and conduct of the study, in analysis and drafting the paper. EYK supported the project and made it feasible and PF ensured quality, participated in the writing of the paper and in the internal committee. PB, SA and JvR participated in the conception of the research, as well as in terms of on-going feed-back and reviewing for analysis and writing. Each author has given final approval of the version to be published.
